# Associations of nutritional status, sugar and second-hand smoke exposure with dental caries among 3- to 6-year old Malaysian pre-schoolers: a cross-sectional study

**DOI:** 10.1186/s12903-020-01152-0

**Published:** 2020-06-03

**Authors:** Zhi Ling Lee, Wan Ying Gan, Poh Ying Lim, Ruhaya Hasan, Sing Ying Lim

**Affiliations:** 1 Department of Nutrition and Dietetics, Faculty of Medicine and Health Sciences, Universiti Putra Malaysia, UPM, 43400 Serdang, Selangor Darul Ehsan Malaysia; 2 Department of Community Health, Faculty of Medicine and Health Sciences, Universiti Putra Malaysia, UPM, 43400 Serdang, Selangor Darul Ehsan Malaysia; 3 School of Dental Sciences, Health Campus, Universiti Sains Malaysia, 16150 Kubang Kerian, Kelantan Malaysia; 4 Department of Paediatric Dental, Tuanku Ja’afar Hospital, Jalan Rasah, Bukit Rasah, 70300 Seremban, Negeri Sembilan Malaysia

**Keywords:** Pre-schoolers, Dental caries, Oral health, Stunting, Second-hand smoke exposure, Sugar exposure, Malaysia

## Abstract

**Background:**

Dental caries in primary teeth is a serious oral health concern among children. It can lead to detrimental impacts on a child’s growth, development, and quality of life. Therefore, this cross-sectional study aimed to examine the prevalence of dental caries and its associations with nutritional status, sugar and second-hand smoke exposure among pre-schoolers.

**Methods:**

A total of 26 pre-schools in Seremban, Malaysia were randomly selected using the probability proportional to size sampling. Dental examination was performed by a dentist to record the number of decayed teeth (dt). Weight and height of the pre-schoolers were measured. The mother-administered questionnaire was used to gather information pertaining to the sociodemographic characteristics and second-hand smoke exposure. Total sugar exposure was calculated from a 3-day food record.

**Results:**

Among the 396 participating pre-schoolers, 63.4% of them had at least one untreated caries, with a mean ± SD dt score of 3.56 ± 4.57. Negative binomial regression analysis revealed that being a boy (adjusted mean ratio = 1.42, 95% CI = 0.005–0.698, *p* = 0.047), exposed to second-hand smoke (adjusted mean ratio = 1.67, 95% CI = 0.168–0.857, *p* = 0.004) and those who had more than 6 times of daily total sugar exposure (adjusted mean ratio = 1.93, 95% CI = 0.138–0.857, *p* = 0.013) were significantly associated with dental caries among pre-schoolers.

**Conclusion:**

A high prevalence of dental caries was reported in this study. This study highlights the need to reduce exposure to second-hand smoke and practice healthy eating behaviours in reducing the risk of dental caries among pre-schoolers.

## Background

Dental caries in primary teeth is a serious oral health concern in children. It is one of the most common non-communicable diseases in children worldwide, which is usually untreated [1]. In 2015, almost 8% of the children globally were affected by untreated caries in the primary teeth [1]. In order to reduce and prevent dental caries in Malaysia, the water fluoridation programme was implemented in 1971 at the concentration level of 0.7 ppm (ppm), and it was adjusted to 0.5 ppm later in 2004 to prevent fluorosis [2]. The water fluoridation programme covered 75.7% of Malaysia in 2016 [3]. However, the rate of caries decrement was still slow among pre-schoolers. Although there was a notable decrease in the prevalence of dental caries, from 87.1% in 1995 to 76.2% in 2005 among 5-year-old Malaysian children, the dft index (number of decayed and filled primary teeth) did not change much throughout the years from 5.8 in 1995 to 5.5 in 2005 [4]. Furthermore, the mean number of the decayed teeth remained high (5.7 to 5.3) but the mean number of the filled teeth remained low at 0.2 from year 1995 to 2005, indicating that most of the caries were untreated [4]. Early treatment of dental caries is needed to address the issue of untreated caries among pre-schoolers in order to improve their oral health.

Dental caries in primary teeth can be a devastating disease if it is not treated properly. It not only affects oral health, but children with caries also experience difficulties in eating and sleeping due to the unbearable pain and soreness, premature tooth loss, disrupt speech development and eventually dental caries in primary teeth can affect the growth and maturation of permanent teeth [5, 6]. In some cases, dental caries may end up fatal [7]. Undoubtedly, dental caries is a major oral health issue in childhood that warrants urgent attention. In Malaysia, while studies on caries have mainly focused on school-aged children [8, 9], there is a dearth of published information on pre-schoolers.

Dental caries is a multifactorial disease involving various risk and protective factors. It is crucial to manage the occurrences of multiple factors during pre-school years to maintain the caries balance, and therefore, prevent dental caries. Sociodemographic factors such as sex, birth order, parental educational level or household income could play a role in the development of dental caries [10, 11]. Previous studies have shown that children with underprivileged social background and experiencing poverty have higher risk of dental caries as compared to those with high socioeconomic status [7, 12]. High sugar exposure (frequency of sugary food and drinks) was found to be another risk factor for caries [13, 14]. Besides that, second-hand smoke brings harm to children’s general heath, including dental health. It is suggested that the exposure to second-hand smoke at home increases the risk of caries among children [12, 15], but some studies found no association [16, 17]. Moreover, obesity and caries are health problems that exist concurrently, mostly due to their common risk factors, including consumption of food high in calories, increased stress, and low socioeconomic status [18]. However, the association of nutritional status and dental caries was inconclusive as some of the studies found positive association [19, 20] while other studies found negative association [21–24].

Most of the previous studies on children used the categorised dmft index (number of decayed, missing, and filled teeth) as the outcome measurement for dental caries. Nonetheless, it failed to capture the severity and the chronic nature of dental caries (decayed teeth). It was assumed that treated caries does not cause burden like untreated caries [25]. The problem and burden of the decayed teeth among children with caries might be masked when dmft index was used to determine their past and current caries experience. Dimaisip-Nabuab et al. [23] suggested that the number of decayed teeth (dt) might be a stronger determinant than the dmft index, when it comes to determining factors such as nutritional status, that are mutually chronic in nature. Therefore, dt score was used in this study to determine dental caries.

As a developing country with a multicultural background, it can be assumed that there might have been some risk factors that are unique to Malaysian pre-schoolers. Findings from the western populations might not be applicable to the Malaysian population due to the different socio-cultural backgrounds, lifestyle, and food intake. Furthermore, dental caries could be one of the indicators for detecting inadequate nutrition in pre-schoolers. Hence, this study aimed to examine the prevalence of dental caries and its associations with nutritional status, sugar and second-hand smoke exposure among Malaysian pre-schoolers.

## Methods

### Selection of participants

This cross-sectional study was carried out from April to November 2018 at government pre-schools in Seremban district of the state of Negeri Sembilan, Malaysia. As children with low socioeconomic status are prone to dental caries [7, 12], this study was conducted at government pre-schools. One of the enrolment criteria set by the Department of National Unity and Integration for the government pre-schools is to take in children with monthly household income of less than MYR1500 (≈ USD 367).

The estimated sample size of 351 was calculated using one proportion formula according to the caries prevalence of 64.9% in Malaysian pre-schoolers [26], considering precision of 0.05 and confidence level of 95%. The probability proportional to size sampling technique was used. A list of 56 government pre-schools in Seremban district and the estimated number of pre-schoolers aged 3 to 6 years old in each pre-school were obtained from the Department of National Unity and Integration. A total of 26 pre-schools were randomly selected. All mothers and pre-schoolers in these selected pre-schools were invited to join the study (*N* = 611). Children with health complications and developmental disability tend to have unmet dental needs compared to typically developing children [27]. Hence, those Malaysian pre-schoolers with any medical complications, developmental delay, and learning disabilities were excluded from this study. A total of 587 out of 611 pre-schoolers met the inclusion criteria of this study. Nevertheless, 165 mothers did not sign the consent form. Hence, 422 pre-schoolers with a response rate of 71.9% were recruited in this study. A total of 396 pre-schoolers and their mothers completed all measures and were included in the final quantitative analysis.

Ethics approval was sought from the Ethics Committee for Research Involving Human Subjects Universiti Putra Malaysia (Reference No.: JKEUPM-2018-043). Permissions to conduct the study were obtained from the Oral Health Division, Ministry of Health and the Department of National Unity and Integration, Malaysia.

### Measures

All mothers of the pre-schoolers answered a set of Malay language self-administered questionnaire at home, consisting of information on sociodemographic characteristics and household smoke exposure. Mothers also completed a 3-day food record of their children at home. Anthropometric measurements were assessed by the researchers at the preschools.

#### Mother-administered questionnaire

Sociodemographic characteristics of the children and their parents were self-reported by the mothers, including child’s age, sex, ethnicity, birth weight, birth order, parents’ age, parental educational level, employment status, marital status, and monthly household income.

Mothers were required to answer “*the number of people who smoke inside the house*” according to the options provided: zero, one, two, three or more, while “*the number of cigarettes smoked inside the house per day*” was asked and the options provided were “never smoked inside the house” or “at least one cigarette smoked inside the house per day” [12].

Furthermore, mothers were required to fill in a 3-day food record (two weekdays and one weekend) regarding their child’s dietary intake. Mothers were requested to record detailed description of food and beverages consumed by their child at home, including the cooking methods, quantity of food and brand names of the processed food as well as the portion size of food and beverages based on the provided photographs of household measurements in the questionnaire. Food and beverages consumed at preschools were obtained from teachers. The total sugar exposure was obtained from the 3-day food record. Total sugar exposure is the frequency of liquid and solid sugar consumption in a day [28]. The mean total sugar exposure was calculated by dividing the total sugar exposure in 3 days by three (number of days) [29]. The total sugar exposure was then classified into two categories: ≤ 6 times daily and > 6 times daily [29].

#### Anthropometric measurements

Height and weight of the pre-schoolers were measured by the researchers using a SECA portable stadiometer 213 (SECA, Hamburg, Germany) and a TANITA digital weighing scale HD319 (TANITA Corporation, Arlington Heights, IL, USA), respectively, with the pre-schoolers standing in upright position, light clothing and without any foot wear. The measurements were recorded in duplicate to get an average of the two readings for analysis. Calibration of the instruments was done by the researcher at the beginning and end of each assessment day. The weight-for-age z score (WAZ), height-for-age z score (HAZ) and BMI-for-age z score (BAZ) were calculated using the WHO AnthroPlus software version 1.0.4 (WHO, Geneva, Switzerland). The classifications of WAZ, HAZ and BAZ were based on the WHO Child Growth Standard [30] for children aged 60 months and below, and the WHO Growth Reference [31] for children aged above 60 months. Children with HAZ and WAZ less than -2SD were classified as stunted and underweight, respectively. Children aged 60 months and below with BAZ above +2SD [30] and those aged above 60 months with BAZ ≥ +1SD were classified as overweight and obese [31].

#### Dental examination

The dental examination was carried out by a dentist in knee-to-knee position using single-use probe and a mirror under good lighting in the classroom. Gauze was used to dry the teeth. The number of decayed teeth in the primary teeth was recorded as the dt (d = decayed, t = teeth) index based on the WHO 1997 criteria to score dental caries [32]. The diagnostic criteria of decayed teeth included a lesion in pit, fissure, or on a smooth tooth surface, a detectably softened floor or wall, undermined enamel or an obvious cavity. If a filled tooth was decayed, it was recorded as decayed teeth. The number of decayed teeth indicated the number of untreated caries in the oral cavity. The Kappa coefficient of intra-examiner reliability was 0.815 in this study.

### Statistical analysis

Data were analysed using IBM SPSS Statistics 24 (IBM SPSS Statistic, Inc., Chicago, IL, USA). Descriptive analysis was performed and the results were presented in the form of frequencies and percentages for categorical variables. On the other hand, the means and standard deviations were presented for continuous variables. As the count variable (dt index) was over-dispersed, negative binomial regression analysis was performed in this study. All the independent variables were initially evaluated separately using univariate negative binomial regression analysis, and the risk factors with *p* < 0.10 were subsequently included in the multivariate negative binomial regression model. Backward model selection was used to determine the risk factors of dental caries in the final model. The statistical significance level was set at *p* < 0.05.

## Results

A total of 396 mother-child dyad participated in this study with the majority of the pre-schoolers were boys (50.5%), Malay (77.5%), and 5 years of age (50.5%). Two-third of the fathers (65.8%) and mothers (68.4%) completed secondary education (Table 1). The prevalence of stunting, underweight and overweight/obesity were 6.9, 11.8, and 14.9%, respectively (Table 2). Half of the pre-schoolers (51.4%) were exposed to second-hand smoke daily. One in 10 of the pre-schoolers (10.6%) had more than six times of sugar exposure regularly. Approximately two-third of the pre-schoolers (63.4%) had at least one untreated caries, with a mean ± standard deviation (SD) dt score of 3.56 ± 4.57.

**Table 1:** Sociodemographic characteristics of pre-schoolers and their parents (*n* = 396)

Variables	n (%)	Mean ± SD
Sex		
Female	196 (49.5)	
Male	200 (50.5)	
Age (years)		5.50 ± 0.62
3	4 (1.0)	
4	91 (23.0)	
5	200 (50.5)	
6	101 (25.5)	
Ethnicity		
Malay	307 (77.5)	
Chinese	47 (11.9)	
Indian	33 (8.3)	
Other	9 (2.3)	
Birth order		
1	108 (27.3)	
2 and above	288 (72.7)	
Father’s age (years) (*n* = 376)		37.87 ± 6.32
Mother’s age (years) (*n* = 376)		34.80 ± 5.10
Father’s educational level (*n* = 380)		
No formal education	3 (0.8)	
Primary education	23 (6.0)	
Secondary education	250 (65.8)	
Certificate/Diploma/A-level	86 (22.6)	
Tertiary education	18 (4.8)	
Mother’s educational level (*n* = 393)		
No formal education	5 (1.3)	
Primary education	18 (4.6)	
Secondary education	269 (68.4)	
Certificate/Diploma/A-level	79 (20.1)	
Tertiary education	22 (5.6)	
Marital status		
Single/Divorced/Separated/Widowed	24 (6.1)	
Married	372 (93.9)	
Monthly household income^a^ (*n* = 392)		
Below MYR4000	343 (87.5)	
MYR4000 and above	49 (12.5)	

^a^Ministry of Economic Affairs [33]; MYR = Ringgit Malaysia; USD 1 = MYR 4.09 as on 31 January 2020

**Table 2:** Dental caries, nutritional status, sugar and second-hand smoke exposure of the pre-schoolers (*n* = 396)

Variables	n (%)	Mean ± SD
Number of decayed teeth in the primary teeth (dt score)		3.56 ± 4.56
dt = 0	145 (36.6)	
dt ≥ 1	251 (63.4)	
Weight-for-age z score (WAZ)		−0.55 ± 1.43
Underweight	46 (11.8)	
Normal	343 (88.2)	
Height-for-age z-score (HAZ)		−0.60 ± 1.03
Stunted	27 (6.9)	
Normal	362 (93.1)	
BMI-for-age z-score (BAZ)		−0.29 ± 1.50
Wasted/Thinness	29 (7.5)	
Normal	302 (77.6)	
Overweight	32 (8.2)	
Obesity	26 (6.7)	
Number of smokers at home (*n* = 395)		
0	148 (37.5)	
1	215 (54.4)	
2	24 (6.1)	
≥ 3	8 (2.0)	
Number of cigarettes smoked inside the house in a day (*n* = 243)		
Never smoked inside the house	118 (48.6)	
≥ 1 cigarette smoked inside the house per day	125 (51.4)	
Total sugar exposure (*n* = 329)		4.45 ± 1.46
≤ 6 times	294 (89.4)	
> 6 times	35 (10.6)	

Variables with *p* < 0.10 were tested in the negative binomial regression analysis. These variables were sex, birth order, WAZ, HAZ, number of cigarettes smoked inside the house in a day, and the total sugar exposure (Table 3). The negative binomial regression analysis showed that sex, second-hand smoke exposure, and the total sugar exposure were significantly associated with the dt index among pre-schoolers (Table 4). Boys had a higher dt score when compared to girls (adjusted mean ratio = 1.43, 95% CI = 1.024–2.006, *p* = 0.036). Pre-schoolers who lived in the household with at least one cigarette smoked inside the house daily had a higher dt score when compared to those who never expose to cigarette smoke inside the house (adjusted mean ratio = 1.61, 95% CI = 1.153–2.260, *p* = 0.005). Pre-schoolers who consumed > 6 times of sugary food or drinks in a day had a higher dt score than those who consumed sugary food or drinks ≤6 times in a day (adjusted mean ratio = 2.09, 95% CI = 1.253–3.483, *p* = 0.005).

**Table 3:** Univariate negative binomial regression analysis of the potential risk factors for decayed teeth (dt) of the pre-schoolers

Variables	B	SE	Mean ratio (95% CI)	*p* value
Sex				
Female^a^				
Male	0.486	0.114	1.63 (1.300–2.035)	< 0.001
Age	0.062	0.899	1.06 (0.892–1.269)	0.490
Birth order	0.087	0.427	1.09 (1.003–1.187)	0.041
Father’s age	−0.004	0.009	1.00 (0.979–1.014)	0.677
Mother’s age	−0.003	0.106	1.00 (0.976–1.018)	0.779
Father’s educational level				
Secondary education and below^a^				
Tertiary education	0.197	0.129	1.22 (0.945–1.567)	0.128
Mother’s educational level				
Secondary education and below^a^				
Tertiary education	0.175	0.130	1.19 (0.924–1.536)	0.176
Marital Status				
Single/Divorced/Separated/Widowed^a^				
Married	0.153	0.242	1.17 (0.725–1.873)	0.527
Monthly household income				
Below MYR4000^a^				
MYR4000 and above	0.076	0.172	1.08 (0.771–1.510)	0.659
Weight-for-age z-score				
Normal^a^				
Underweight	0.379	0.173	1.46 (1.041–2.052)	0.029
Height-for-age z-score				
Normal^a^				
Stunted	0.618	0.213	1.86 (1.224–2.814)	0.004*
BMI-for-age z-score				
Underweight^a^				
Normal	0.346	0.228	1.41 (0.905–2.209)	0.128
Overweight	−0.850	0.303	0.92 (0.507–1.663)	0.778
Obese	0.332	0.311	1.39 (0.758–2.564)	0.285
Number of cigarettes smoked inside the house in a day				
Never smoked inside the house^a^				
≥ 1 cigarette smoked inside the house per day	0.358	0.146	1.43 (1.076–1.903)	0.014*
Total sugar exposure				
≤ 6^a^				
> 6	0.666	0.196	1.95 (1.326–2.857)	0.001*

^a^Reference group, *B* log (dt index), *SE* Standard Error; *p < 0.05

**Table 4:** Multivariate negative binomial regression analysis of the potential risk factors for decayed teeth (dt) of the pre-schoolers

Variables	B	SE	Adjusted mean ratio (95% CI)	*p* value
Sex				
Female^a^				
Male	0.360	0.172	1.43 (1.024–2.006)	0.036*
Number of cigarettes smoked inside the house in a day				
Never smoked inside the house^a^				
≥ 1 cigarette smoked inside the house per day	0.479	0.172	1.61 (1.153–2.260)	0.005*
Total sugar exposure				
≤ 6 times^a^				
> 6 times	0.737	0.261	2.09 (1.253–3.483)	0.005*

AIC value = 853.49, AICC value = 854.10, BIC value = 876.37

^a^Reference group, *B* log (dt index), *SE* Standard Error; *p < 0.05

## Discussion

Due to the limited study on dental caries in Malaysian pre-schoolers, this study would provide fundamental for the planning and implementation of community programmes among pre-schoolers. This study showed that 63.4% of the pre-schoolers had at least one decayed tooth in their primary teeth. Several studies in Malaysia had revealed the prevalence of dental caries in primary teeth ranging from 44.6% in 7 to 11 years old children [8] to 98.1% in 5 to 6 years old pre-schoolers [24]. School children in the age group of 7 to 11 years had mixed dentition, indicating they had primary teeth and permanent teeth at the same time. The primary teeth fall off gradually and permanent teeth erupted over time, this could lead to lower prevalence of caries in primary teeth among school children [8]. Prevalence of dental caries in the current study was comparable to the prevalence reported in other studies from developing countries such as Brazil (67.7% in pre-schoolers aged 3 to 4 years) [13] and Ecuador (65.4% in pre-schoolers aged 6 years and below) [5]. Meanwhile, the caries prevalence in the current study was much higher in comparison to the prevalence in developed countries such as United States (23.0% in pre-schoolers aged 2 to 5 years old) [34] and Greece (10.0% in pre-schoolers aged 2.5 to 5.9 years) [20]. However, the caries prevalence in the current study was lower than the prevalence shown in other Southeast Asian studies such as in Indonesia (90% in children aged 5 to 12 years) [35] and Vietnam (88.3% in pre-schoolers aged 4 years) [10]. The prevalence in this study was comparable with the prevalence in a national survey (64.9%) conducted in 2015 on pre-schoolers in Malaysia [26]. This might be a result of the increased public awareness towards dental caries, and the strategies implemented by the Oral Health Division in Ministry of Health Malaysia to improve oral health of pre-schoolers by providing maximum coverage via Primary Oral Healthcare programmes targeted at toddlers and pre-schoolers. Nevertheless, the comparison between the prevalence should be done cautiously because these studies showed differences in the sociodemographic characteristics of the children, differences in methodology approaches and difference definitions of caries were used.

This study found a significant association between sex of the pre-schoolers and their dental caries’ experiences. In general, males exhibited more decayed primary teeth in comparison to females. This finding corroborated with the results reported in other studies conducted in Southeast Asia [10, 36]. Biological factors such as earlier eruption and longer retention of primary teeth among boys, as well as psychological factors such as the innate refusal to compliance in boys [37] might be the reasons of higher prevalence of caries among boys. However, this finding was inconsistent with a study done in India, in which they found that Indian girls aged 12 to 15 years old reported higher caries than boys [38]. One of the possible reasons might be due to the shift of caries risk from males to females among older children. Such a shift would have eventually resulted from biological factors such as hormonal changes during girls’ puberty or cultural factors such as less medical attention given to girls in the Indian families [39].

The present study found that HAZ, WAZ or BAZ, was not associated with untreated caries in the primary teeth of pre-schoolers. This supported the findings of several previous studies, which exhibited no relationship between nutritional status and dental caries among children [40–42]. The null association between obesity and caries could be explained by the chronic nature of both diseases. The association between obesity and dental caries might have taken several years to establish, which the effect might be more profound on the permanent teeth of the older children [43]. However, the results were inconsistent with studies from developed countries such as Sweden [19] and Greece [20], which reported positive associations between nutritional status and dental caries among children. A recent systematic review reported similar trend in which high occurrences of caries were found among overweight or obese children from high-income countries, but not children from low- or middle-income countries [44]. The development of dental caries in children from developed countries might undergo different pathway compared to that of children from developing countries [45]. Further investigations are needed to understand the differences in the effect of nutritional status on the development of dental caries between developed and developing countries.

The present study revealed that the second-hand smoke exposure was associated with untreated caries in the primary teeth among pre-schoolers, which was in agreement with the findings from previous studies [12, 15, 46]. A meta-analysis reported that the children who were exposed to second-hand smoke during infancy had 1.72 times higher risk of having caries in their primary teeth than the children who were not exposed [46]. The association of second-hand smoke exposure and dental caries could be explained through one of the toxins found in the smoke: nicotine. Experimental evidence supports a positive association between second-hand smoke exposure and dental caries. An in vivo study found that nicotine increased the attachment of cariogenic bacteria, *Streptococcus mutans* on the nicotine-treated rats’ teeth surfaces [47]. Elevated level of *Streptococcus mutans* was found in human who exposed to cigarette smoke. High amount of *Streptococcus mutans* can secrete more extracellular polysaccharides that lower the pH value of the biofilm on the surfaces of the teeth, which in turn increased demineralization [47]. Furthermore, second-hand smoke decreased the mineralisation of tooth, the rate of salivary flow, and impaired immunity, which led to the colonisation of cariogenic bacteria in teeth [48].

Despite these possible mechanisms, González-Valero et al. [46] suggested that second-hand smoke exposure and dental caries could share similar sociodemographic and behavioural factors. Jakhete and Gitterman [49] found that exposure to second-hand smoke and poor nutrition increased the risk of dental caries in children from low socioeconomic background. Mattheus et al. [12] showed that parents who smoked might have poor oral health and high number of bacteria in the oral cavity, which could then be transmitted to their children through shared eating utensils. Hence, it is important for researchers to distinguish that second-hand smoke could be a risk factor that comes with unhealthy behaviour or an enhancing factor that increases the risk of caries development among pre-schoolers.

The significant and positive association of sugar exposure with dental caries was supported by previous studies [13, 14], which found that a high frequency of liquid or solid sugar consumption was significantly associated with dental caries in pre-schoolers. The association supports the hypothesis proposed by Stephan [48]. The introduction of fermentable carbohydrates to oral cavity reduced the salivary pH beyond the critical pH value of 5.5 in 5 to 10 min, due to the acidic by-products produced by cariogenic bacteria such as *Streptococcus mutans* [48]. It usually takes 30 to 40 min for saliva to neutralize the acid. Nevertheless, the high frequency of sugar exposure increased the frequency of drop in salivary pH. The acidic environment in the oral cavity dissolved the surfaces of the teeth and made the teeth vulnerable to caries [48].

In general, findings of the current study would help dental professionals and policy makers in planning dental care services for pre-schoolers. It is important to have routine oral check-up by dentists or nurses among pre-schoolers in pre-schools. Findings of this study would also serve as a guide in developing appropriate dental caries prevention strategies or oral health promotion programmes for pre-schoolers. Furthermore, educating parents about healthy eating and lifestyle is also important in order to improve oral health of pre-schoolers.

Several limitations of this study should be addressed. This study was a cross-sectional study in which the causal relationship between the factors and dental caries could not be established. Cohort study is necessary to establish the relationships between sugar exposure, second-hand smoke exposure and nutritional status with dental caries in children. This study was carried out among pre-schoolers in government pre-schools in one state in Malaysia, which is not representative to all pre-schoolers in Malaysia. Hence, the findings could not be extrapolated to all Malaysian pre-schoolers. Furthermore, the food record might be subjected to biasness as mothers might tend to provide socially desirable answer, and not record sweet foods and drinks consumption. As pre-schoolers have certain extent of autonomy over their food intake, mothers might not be able to accurately record every single food or drinks consumed by their child. Another limitation of this study was that the second-hand smoked exposure, which is known to be harmful, may be under-reported by the mothers. Future studies could include other more accurate methods in assessing second-hand smoke exposure including biomarkers such as serum cotinine level or hair nicotine level. Furthermore, this study did not include oral health behaviours of children, which is considered as one of the significant risk factors of dental caries. Oral health behaviours such as toothbrushing practice and dental visits could be possible factors that mitigate the effects of sugar exposure on primary teeth of pre-schoolers.

## Conclusions

The prevalence of dental caries among pre-schoolers in this study was high. The results indicate that being a male, exposed to second-hand smoke and having more than 6 times of sugar exposure daily increased the risk of dental caries in pre-schoolers. Prevention strategies such as education regarding sugar exposure and community smoke cessation program should be incorporated into the existing oral health care system.

## Data Availability

The datasets used and/or analysed during the current study are available from the corresponding author on reasonable request.

## Abbreviations

BMI: Body mass index
BAZ: BMI-for-age Z score
CI: Confidence interval
dft: Number of decayed and filled teeth in the primary teeth
dt: Number of decayed primary teeth
HAZ: Height-for-age Z score
MYR: Ringgit Malaysia
SD: Standard deviation
SE: Standard error
WAZ: Weight-for-age Z Score
WHO: World Health Organization
